# Active learning-based machine learning approach for enhancing environmental sustainability in green building energy consumption

**DOI:** 10.1038/s41598-024-70729-4

**Published:** 2024-08-27

**Authors:** Shahid Mahmood, Huaping Sun, Amel Ali Alhussan, Asifa Iqbal, El-Sayed M. El-kenawy

**Affiliations:** 1 School of Finance and Economics, Jiangsu University, Zhenjiang, China; 2 School of Economics and Management, University of Science and Technology Beijing, Beijing, 100083 China; 3 Department of Computer Sciences, College of Computer and Information Sciences, Princess Nourah bint Abdulrahman University, P.O. Box 84428, 11671 Riyadh, Saudi Arabia; 4 School of International Studies, Zhengzhou University, Zhengzhou, China; 5 Department of Communications and Electronics, Delta Higher Institute of Engineering and Technology, Mansoura, 35111 Egypt; 6 MEU Research Unit, Middle East University, Amman, 11831 Jordan

**Keywords:** Artificial intelligence, Energy consumption, Environmental sustainability, Green building, Machine learning, Environmental social sciences, Environmental sciences

## Abstract

Green building (GB) techniques are essential for reducing energy waste in the construction sector, which accounts for almost 40% of global energy consumption. Despite their importance, challenges such as occupant behavior and energy management gaps often result in GBs consuming up to 2.5 times more energy than intended. To address this, Building Automation Systems (BAS) play a crucial role in enhancing energy efficiency. This research develops a predictive model for GB design using machine learning to minimize energy consumption and improve indoor sustainability. The dataset is utilized to predict cooling and heating individually, with data visualization by graphically illustrating dataset features and preprocessing through Z-Score normalization and dataset splitting. The proposed model, based on active learning and utilizing ML regressors such as Random Forest (RF), Decision Tree (DT), Gradient Boosting (GB), Extreme Gradient Boosting (XGBoost), CatBoost (CB), Light Gradient Boosting Machine (LGBM), K-Nearest Neighbor (KNN), and Logistic Regressor (LR), shows significant performance improvements. The CBR-AL model achieves impressive results with values of 0.9975 for cooling (Y1) and 0.9883 for heating (Y2), indicating a high level of accuracy. The model’s success in reducing energy consumption and improving sustainability has potential ripple effects, including substantial cost savings, reduced carbon footprints, and improved operational efficiency in green buildings. This approach not only enhances environmental sustainability but also sets a benchmark for future advancements in predictive modelling for energy management.

## Introduction

The global pursuit of sustainability greatly depends on the built environment. Green buildings are a leading indicator of achievement in this attempt because of their low environmental impact and effective use of resources. To advance the sustainability agenda and guarantee an economically viable and sustainable future for communities globally, it is imperative to integrate cutting-edge technologies with the principles of green building^1^. The concept of sustainable development in the built environment is the idea of designing environment that includes human activity with the limited use of the resources, limited environmental degradation and more sustainable communities. Since the built environment is mostly responsible for the world’s energy consumption and carbon emissions, transforming it into a sustainable state is essential to reducing global warming and preserving ecological integrity for future generations^2^. Environmental sustainability aims for building less vulnerable communities, by designing for passive heating and cooling and installing green infrastructure, which can promote economic development and job creation without harming the environment^3^.

Green buildings become essential catalysts for reducing the built environment’s environmental impact. Green buildings are characterized by their integrated use of renewable energy sources, energy-efficient technologies, and sustainable design concepts to reduce waste production, water consumption, energy consumption, habitat preservation and indoor environmental quality optimization^4^. Green buildings lessen dependency on fossil fuels, reduce greenhouse gas emissions, and ease the burden on natural resources by utilizing techniques like passive solar design, effective insulation, and natural ventilation. Additionally, they improve occupant comfort, provide healthier interior settings, and encourage well-being, highlighting the mutually beneficial relationship between environmental sustainability and human health by utilizing water, energy, and other resources minimally and in a balanced manner^5^.

Subsets of artificial intelligence (AI) known as machine learning (ML) and deep learning (DL) allow computers to learn from data and make predictions or judgments without the need for detailed programming^6^. Machine learning and deep learning are highly versatile and may be applied to a wide range of sectors, encouraging innovation and increasing productivity. In the energy sector, these technologies are employed for tasks like enhancing grid management, optimizing energy consumption, and carrying out equipment preventive maintenance^7^. These technologies are essential to the healthcare sector because they allow for medical image analysis, personalized beneficial recommendations, and illness diagnosis. In finance, algorithmic trading, detection of fraud, and risk management are all enabled by ML and DL^8^. Demand forecasting and recommendation systems driven by these technologies are used by companies in retail to enhance customer experiences and optimize workflows^9^. In the manufacturing industry, ML and DL are utilized for predictive maintenance, quality assurance, and supply chain management. The energy, entertainment, and transportation industries also use this technology for a range of tasks, including developing autonomous autos and projecting energy use. These sectors could use deep learning and machine learning to boost productivity, foster innovation, and enhance operational effectiveness. When applied to green buildings, these techniques have the potential to increase energy efficiency and environmental sustainability significantly.

The easy incorporation of renewable energy sources made attainable by ML and DL technology enhances the energy ecosystem of green buildings. Through the application of predictive modeling and optimization algorithms, these technologies maximize the use of solar, wind, and other sustainable energy sources, reducing reliance on conventional power sources and promoting energy liberty. Ghazi Mirza ML and DL enable the deployment of occupant-centric controls and comfort techniques in green buildings. Real-time improvements in comfort, productivity, and general well-being are made possible by these technologies, which also help to achieve energy efficiency targets. To achieve this, they analyze utilization, temperature preference, and activity level data. Modeling and simulating dynamic building performance requires the use of ML and DL models. Through the delivery of guidance for remodeling efforts, the optimization of operational methods, and the evaluation of big datasets to predict building performance under various conditions, these technologies boost the effectiveness of green building initiatives and foster continuous development^10^ in his building integration of ML and DL technologies, allows green buildings to optimize energy efficiency, minimize environmental impact, and enhance occupant well-being a significant step towards a more sustainable built environment.

Machine learning methods such as Random Forests and Support Vector Machines (SVM) have been applied to predictive maintenance in green buildings. By analyzing sensor data from building systems and equipment, these algorithms anticipate potential equipment failures and enable proactive maintenance schedules. However, real-time applications might not always have access to vast volumes of historical data. Thus hybrid models that combine physics-based simulations with machine learning must be developed in order to improve forecast accuracy and reliability^11^.

DL models such as convolutional neural networks (CNN) and long short-term memory (LSTM) networks are vital for enhancing energy efficiency in green buildings. These models analyze historical data on energy usage, ambient conditions, and utilization patterns to dynamically adjust building systems to minimize energy waste and maintain occupant comfort. However, in order to address computational complexity, it might focus on developing lightweight deep learning architectures for energy optimization tasks, which would enable faster inference and deployment on edge devices^12^. Algorithms for machine learning-based reinforcement learning, such as Proximal Policy Optimization (PPO) and Deep Q-Networks (DQN), can support occupant-centric control strategies in green buildings. By learning the best control policies through interactions with the building environment and inhabitants, these algorithms are able to optimize building operations to meet occupant preferences and usage patterns^13^. However, the need for extensive, costly, and time-consuming training in real-world contexts is a disadvantage of RL approaches. Future efforts could look into transfer learning techniques to make use of pre-trained RL models and speed up resolution in fresh construction environments^13^. ML approaches, such as Time-Series Forecasting models like Autoregressive Integrated Moving Average (ARIMA) and Recurrent Neural Networks (RNN), are crucial for forecasting the output of renewable energy in green buildings. These models can be effectively included into building energy systems since they can calculate the amount of energy generated from renewable sources. They accomplish this by looking at wind speed, sun irradiance, and historical meteorological data. It does not, however, address traditional forecasting methods based on ML, which are dependent on previous data and cannot detect sudden changes or anomalies. It might focus on fusing real-time data streams with ensemble learning approaches to increase the accuracy and robustness of forecasts for renewable energy^14^.

### Rationale of the study

The potential for increasing efficiency and environmental sustainability in green buildings through the integration of ML and DL approaches is tremendous. AI makes proactive interventions to prevent equipment breakdowns possible, reducing downtime and energy waste by utilizing predictive maintenance algorithms. AI-driven optimization strategies maximize resource efficiency while minimizing environmental effects by facilitating adaptive controls, dynamic energy management, and seamless integration of renewable energy sources^15^. AI enables green buildings to adapt, change, and eventually reach higher levels of sustainability and efficiency through data-driven insights and continuous learning mechanisms. By investigating the methods, uses, and possible drawbacks of AI in green buildings, we hope to establish the foundation for a more resilient and ecologically conscious built environment that balances the requirements of people and the demands of the environment. When evaluating complex interactions uncovered by building data, the ML and DL-based techniques perform better, producing more accurate predictions and optimized energy management plans. This development represents a major step forward in the application of innovative AI methods to improve energy efficiency and boost sustainability in the built environment^16^.

The following are this paper’s primary contributions:

- We propose a technique for green building design by applying active learning-based techniques based on machine learning models that can reduce energy consumption and improve indoor environment impact to enhance environmental sustainability.
- We apply the proposed models to the energy efficiency dataset. We perform data visualization by visualizing features of the dataset, utilizing different graphs and data preprocessing techniques such as feature normalization (Z-Score normalization) and data splitting to normalize the data and divide the data into training and testing sets.
- The experiment’s findings show that the proposed active learning-based technique performs better in terms of prediction accuracy (R^2^), MSE, MAE and RAE. The proposed CBR-AL outperforms all other models with a value of 0.9975 on all iterations.

## Literature review

This section provides previous work related to green buildings for sustainable architecture utilizing traditional, machine, and deep learning techniques for energy consumption and the indoor environment.

### Traditional techniques in green buildings for sustainable architecture

In the building and construction industry, sustainability is becoming increasingly important as a major driver of social, economic, and environmental advantages with fewer negative environmental repercussions. For the industry to become more energy efficient, green and sustainable techniques must be used. This is particularly valid when utilizing the most recent green technology. The goal of the study^17^ is to identify the most useful methods for use in green construction, evaluate the benefits of doing so, and look at the best practices for green building qualities. The study’s findings showed that employing sustainable practices and energy-efficient technologies can result in green buildings with reduced energy consumption as well as cheaper long-term operating and maintenance costs. The aim of the^18^ research is to provide definitions for the words “green building” and “sustainability” in relation to residential building design since it’s critical to understand how sustainable design principles may assist in reducing adverse impacts on society and the environment. The approach of case studies is utilized. Three case studies of green buildings one each from China, Indonesia, and Dubai are examined in this research with an emphasis on the creative and sustainable design aspects used. The results showed that those countries are committed to the creation of ecologically friendly buildings, especially homes, in order to achieve the maximum degree of social, economic, and environmental sustainability.

The author of the^19^ research creates a set of sensible and reliable social sustainability metrics in order to evaluate green buildings in China. Indicators for green buildings are essential for confirming different research findings and assisting professionals in comprehending social sustainability indicators. Consequently, these signals are examined using the fuzzy Delphi approach. The findings indicate that the most crucial elements of socially responsible green design are robustness and security. The following other essential components are: comfort, accessibility, convenience, and health. To attain social sustainability, this set of indicators helps practitioners make decisions and offers pertinent, beneficial advice for a number of stakeholders. The author of^20^ proposed a useful mapping tool that uses green building rating tools (GBRTs) to determine how much a green building contributes to the Sustainable Development Goals (SDGs). It then conducted a quantitative analysis of this contribution using the analytic hierarchy approach. Results showed that GBRTs contribute significantly to SDGs 3, 7, 11, and 12, with SDG 12 benefiting the most. However, SDG Target 7.3 stands out the most since it presents the most opportunity for GBRT to make a meaningful contribution to the SDGs.

The advantages of green building technologies (GBTs) for the environment, the economy, and society at large have led to their notable growth in recent years. The main objective of GBTs is to use energy, water, and other resources sensibly and in a balanced way. As a result, the environment will get better. Green buildings reduce energy consumption and greenhouse gas emissions, improve productivity and health, and save maintenance and operating expenses. The objective of the^21^ study is to pinpoint significant issues in the field of green building research that are related to long-term, cost-effective, environmentally benign, and sustainable building techniques that also consider future advancements. In order to ensure a sustainable future, this paper looks at the current status of green building construction and makes recommendations for further research and development. This study also suggested a few potential paths for future research on sustainable development in an effort to stimulate greater investigation.

### Machine learning techniques in green buildings for sustainable architecture

An extensive budget is necessary, especially for green buildings and other sustainable initiatives. In the construction sector, green building construction contracts are still relatively new, and stakeholders desire more experience with contract cost estimates. Green buildings, as opposed to conventional construction, use state-of-the-art technologies to reduce the negative effects of their operations on society and the environment. In order to estimate the pricing of green buildings, the author of the^22^ study recommends machine learning-based techniques including extreme gradient boosting (XGBOOST), random forest (RF), and deep neural network (DNN). The suggested models are built with the impact of both hard and soft cost-related variables taken into account. The accuracy of the created algorithms is compared and examined using evaluation criteria. With an accuracy of 0.96, XGBOOST outperformed the DNN’s 0.91; RF came in second with 0.87 accuracy.

Buildings need to be sustainable and energy-efficient since they have a significant impact on the world’s energy consumption and greenhouse gas emissions. Utility providers, customers, and facility managers can all benefit from the ability to predict trends of energy usage in buildings since it can lead to increased energy efficiency. The authors of^23^ study optimized and effectively reduced the energy consumption of smart cities using a reinforcement learning approach. The recommended reinforcement learning approach employs a group of agents working together with an optimal energy distribution policy to accomplish a common goal. The capacity of agents to collaborate in order to maximize energy consumption and save costs is only one of the numerous benefits that will result from their coordinated efforts. The proposed method evaluates each choice by analyzing data on energy use and the extent to which the option has been used in the past. This architecture ensures that the device keeps its energy footprint and communication dependability as balanced as possible. The simulation’s results indicate that the smart city’s yearly energy consumption might be reduced by more than 35–40% by optimizing energy usage with the recommended reinforcement learning technique.

Using an hourly resolution forecasting technique based on Random Forests (RF), the short-term energy consumption in different buildings was estimated^24^. The actual energy consumption of buildings was measured over a year using five datasets, which were used to evaluate the effectiveness of the RF model in training and testing. The evaluation’s conclusions showed how well the RF model predicted the future. In terms of mean absolute percentage error (MAPE), 49.21%, and 46.93% in the MAE, the RF model outperformed the RT model when it came to projecting building energy consumption one step ahead of time. The remarkable performance was confirmed by the RF model, which showed improvements in MAE of 49.95% and 29.29% above the M5P model in the 12-steps-ahead and 24-steps-ahead energy usage, respectively. The study^25^ adjusts the thickness and lay-up of Nano-insulation to maximize energy loss in green buildings. Thermal conductivity is successfully predicted by machine learning models such as decision trees, Gaussian Process Regression, and Support Vector Machines. The decision tree model performs better than the others. Energy efficiency is improved by taking into account energy usage, economics, and environmental impact through the use of a multidisciplinary optimization method. The findings demonstrate a 40% increase in energy savings above standard insulation, with reduced CO2 emissions ranging from 290 to 293 kg/m^3^, depending on a number of variables. The impact of DSF segmentation on energy consumption and natural ventilation in high-rise structures in hot and dry regions is examined by the author in the paper^26^. Sixty-four segmentation scenarios were examined in an eight-story residential building in Isfahan, Iran, using DesignBuilder software. HGS-GB, a hybrid model that combines Gradient Boosting and Hunger Game Search, was created to estimate energy usage in a variety of scenarios. High R^2^ values for lighting, heating, cooling, and total energy estimation show that the HGS-GB model performs better than individual models such as Gradient Boosting, Random Forest, and K-nearest neighbors. The results highlight how crucial DSF segmentation is to maximizing ventilation and energy use in these kinds of structures.

### Deep learning techniques in green buildings for sustainable architecture

The study^27^ offers a practical deep-learning approach to mosque energy consumption estimation. A Convolution Neural Network (CNN) deep learning model is developed to predict the annual energy consumption of mosque constructions based on multiple operational scenarios. A thorough energy analysis model with a range of simulation findings and three-dimensional laser scanning is used to build the mosque’s simulated energy consumption dataset. The operating schedule, the mosque’s zone division, the use of dimmers, the existence of a cooling system and its setting point, and the power consumption of the appliances and lighting system are just a few of the operational factors that have been used to estimate the mosque’s annual energy consumption in order to create the database. Using the mosque’s simulated energy consumption dataset, the CNN model was trained and assessed to find the best model configuration based on the given performance criteria. Furthermore, the performance of the produced model was compared to that of the Support Vector Regression (SVR) model. The MAPE and  R^2^ values of the derived model are 4.5% and 0.98%, respectively.

The goal of the study^28^ is to solve incomplete data gathering for building energy consumption (EC) and inadequacies in conventional backpropagation neural networks (BPNN). Applying generative adversarial networks (GANs) to enhance data quality in a green building (GB)-oriented EC data generation model offers a solution. To increase prediction accuracy, an EC prediction model based on Levenberg Marquardt-optimized BPNN is then created. The usefulness of the suggested approach is confirmed with actual building energy consumption data. The optimized BPNN model outperforms other models, providing lower prediction errors and increased performance. The results show that GAN-enhanced data reduces prediction errors. A clustering-based approach to energy consumption analysis is presented by the author in^29^, who divides users’ electricity usage into various tiers. To begin with, low-dimensional energy consumption data is transformed into high-level representations by training a deep autoencoder. After that, an adaptive self-organizing map (SOM) clustering technique is used using these representations as input. Electrical energy consumption levels are then ascertained by statistically analyzing the clustered data. Graphs, calendar views, and city maps are used to show the data, providing energy providers with a thorough perspective to assess and improve energy consumption.

The author of the^30^ study suggests using deep neural network models to optimize building energy usage in Buraydah, Saudi Arabia, which has a severe and arid climate. The models are adjusted according to hyper-parameters like hidden layers, neuron count, and learning algorithms using data produced by simulation software. With MSE of 0.0075, RMSE of 0.087, R and R^2^ of 0.99 for heating load prediction and MSE of 0.245, RMSE of 0.495, R and R^2^ of 0.99 for cooling load prediction, the five-layer model with 20 neurons per layer and the Levenberg–Marquardt method performs better than the others. When constructing energy-efficient buildings, architects can use this data as a helpful resource. In order to increase prediction accuracy and computational efficiency, the paper proposes DF-DQN, a novel technique that combines deep forests with deep Q-networks (DQN)^31^. To improve optimization, DF–DQN uses a reduced version of the original action space, maps it to a single sub-action space using deep forests, and creates new states based on state class probabilities gleaned from deep forests. According to experimental data, DF–DQN with state classes works better than other approaches; in comparison to the DDPG method, R^2^ increases by 0.3% while MAE, MAPE, and RMSE decrease by 5.5%, 7.3%, and 8.9%, respectively.

Author of^32^ explores AI’s role in enhancing energy and resource management efficiency, focusing on prediction, optimization, and automation using Deep Learning techniques such as Convolutional Neural Networks (CNN) and Long Short-Term Memory (LSTM) networks. The findings indicate that AI models significantly improve efficiency and sustainability by providing accurate predictions and automation recommendations, emphasizing the importance of integrating these technologies to optimize energy use and achieve sustainability goals. Traditional methods often rely on expert knowledge and subjective decisions, leading to significant challenges. Recent research by^33^ introduces an innovative framework that combines Building Information Modeling (BIM), Explainable Artificial Intelligence (XAI) using LIME (Local Interpretable Model-agnostic Explanations), and multi-objective optimization (MOO) to address these issues. The framework consists of three main components: data generation through DesignBuilder simulation, a Bayesian optimization–LightGBM (BO-LGBM) predictive model for energy prediction and interpretation, and the multi-objective optimization technique AGE-MOEA to handle uncertainties. A case study demonstrated the framework’s effectiveness, with the BO-LGBM model achieving high prediction accuracy (R-squared > 93.4%, MAPE < 2.13%). LIME successfully identified significant features of the HVAC system, and the AGEMOEA optimization improved energy consumption, CO2 emissions, and thermal comfort by 13.43%, with an additional 4.0% optimization gain when incorporating uncertainties. This study significantly enhances the transparency of machine learning predictions and efficiently identifies optimal passive and active design solutions, contributing to sustainable construction practices.

## Proposed framework

**Fig. 1:**
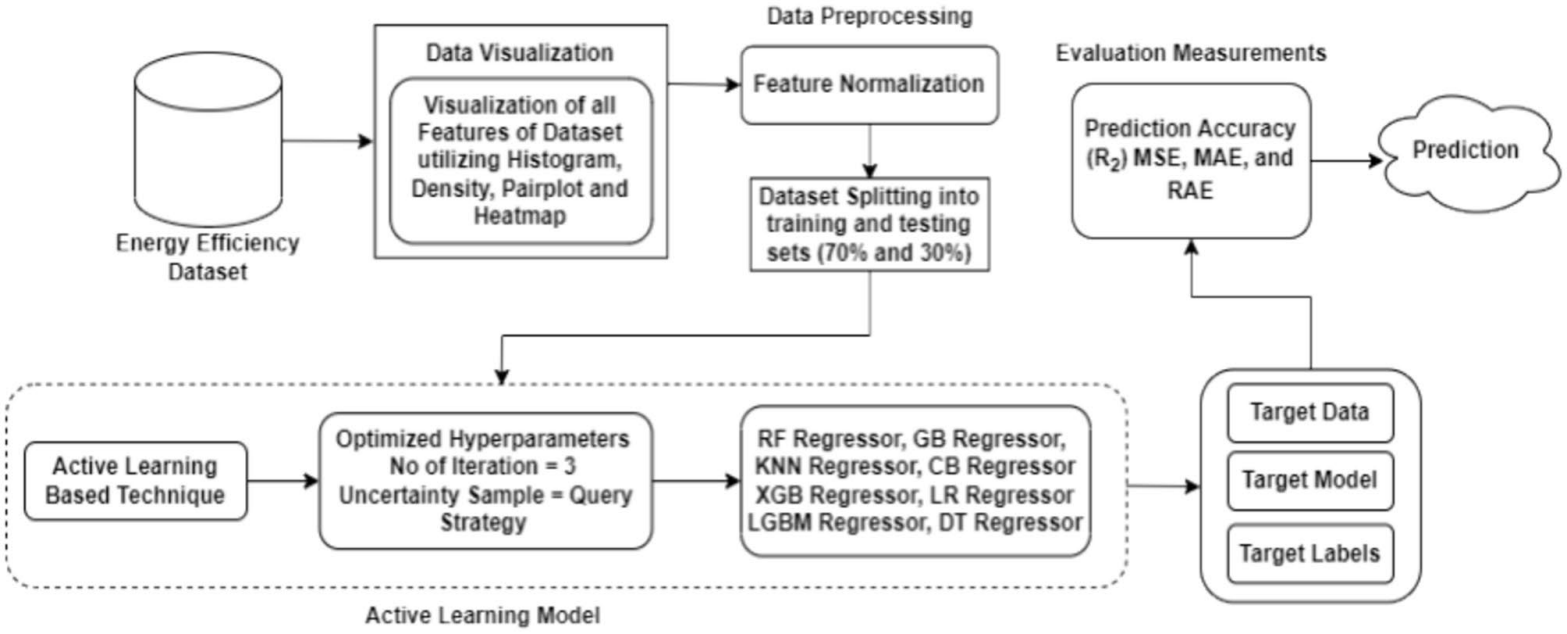
Proposed methodology of active learning model.

This section explains the working methodology of the proposed framework. The objective of this approach is to develop a precise prediction model for energy consumption and indoor environment in green buildings using an active learning model. It encompasses various stages, including dataset visualization, preprocessing, model selection, and the utilization of machine learning techniques for implementation. This process is visually depicted in Fig. 1.

The energy efficiency dataset, which is used for prediction, is the starting point of the suggested system. Data visualization is done by using various graphs, including density, pair plots, and heatmap graphs, to display the features of all datasets. The preprocessing techniques applied to the dataset include feature normalization, which normalizes the data. The model implementation results in the dataset being divided into 70% training data and 30% testing data. Ultimately, a machine learning model based on active learning was taught to identify energy use in green buildings accurately. The ActiveLearner object, implementing our active learning strategy, is constructed with various regressor models. By analyzing the most datasets, our method iteratively queries the energy efficiency class and trains it. Uncertain sampling queries, employing a 3-iteration count for each class, label the samples. The pipeline incorporates eight regressors: random forest, XGBoost, decision tree, logistic regression, gradient boosting, K-nearest neighbor, CatBoost and LightGBM, each creating an Active Learner object to implement the active learning methodology. Subsequently, using prediction accuracy (R^2^), the MSE, MAE and RAE of each regressor are evaluated. For performance comparison, each regressor receives an evaluation dataset and produces a fnal regression report.

### Dataset preliminaries

This study utilized energy efficiency data to identify energy consumption in green buildings. The dataset was generated by Angeliki Xifara and analyzed by Athanasios Tsanas^34^. For energy analysis, Ecotect simulates 12 different building shapes. The buildings differ in their glazing area, glazing area distribution, and building orientation, among other things. Using the earlier specified factors, various situations are simulated to produce 768 building forms. The dataset has 768 samples and 8 features with the goal of predicting two real-valued responses. The dataset consists of two responses (or outcomes, indicated by y1 and y2) and eight qualities (or features, represented by X1–X8) represented in Table 1. The objective is to forecast each of the two states using the eight features.

**Table 1:** Description of dataset features.

Features	Description
X1	Relative compactness
X2	Surface area
X3	Wall area
X4	Roof area
X5	Overall height
X6	Orientation
X7	Glazing area
X8	Glazing area distribution
Y1	Heating load
Y2	Cooling load

**Fig. 2:**
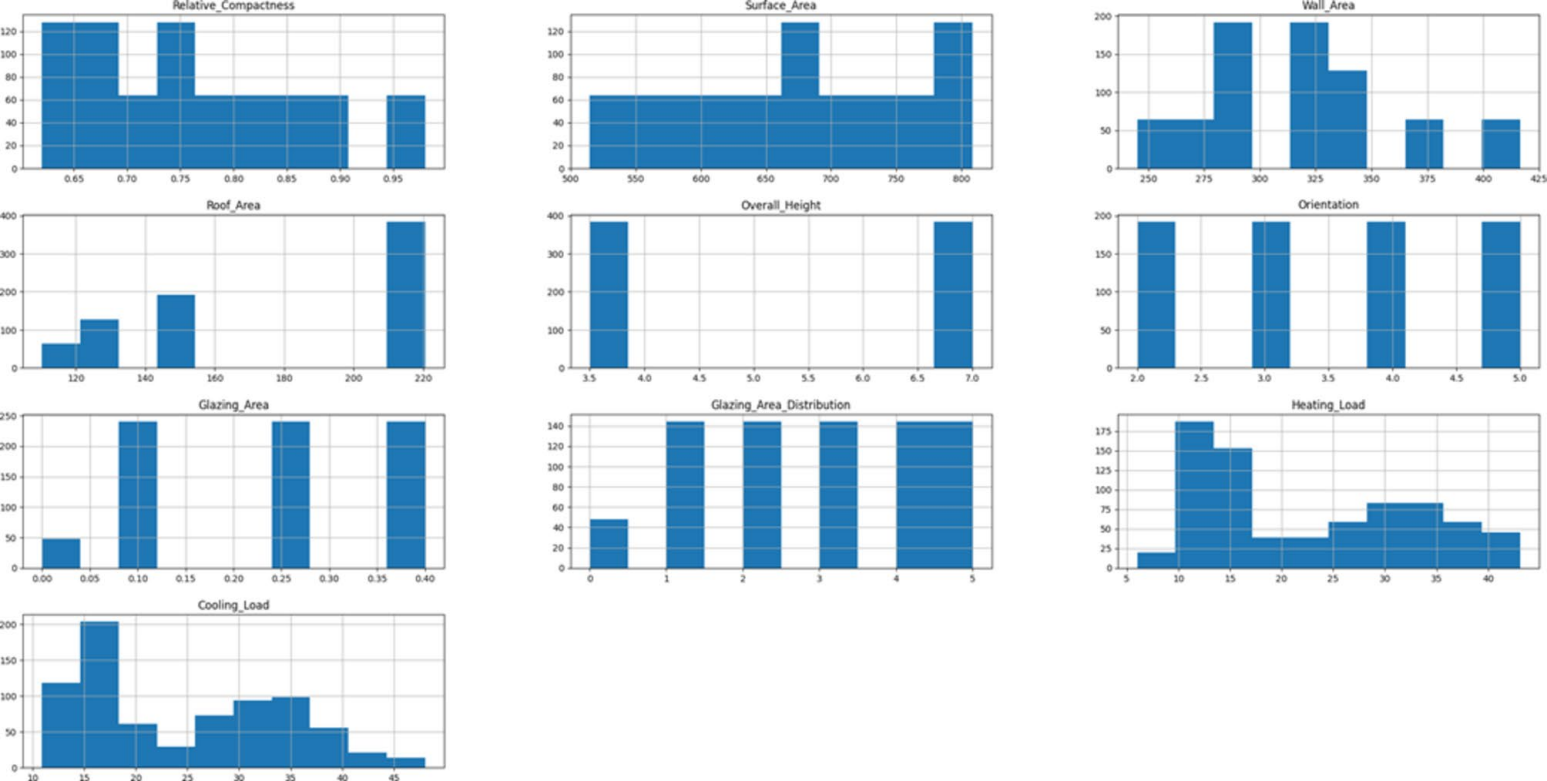
Graphical visualization of histogram for each energy efficiency dataset feature.

Figure 2 presents a graphical display of histograms that show how features related to energy efficiency are distributed across a dataset. Using bars to show the proportion or count of data points inside particular value ranges or bins, histograms are useful tools for visually expressing data distributions. Understanding the distribution of the data, possible skewness, and the existence of outliers is made possible via histogram analysis. The components on the x-axis of energy efficiency probably correspond to various variables of energy consumption, including building characteristics, appliance usage frequency, and environmental conditions.

**Fig. 3:**
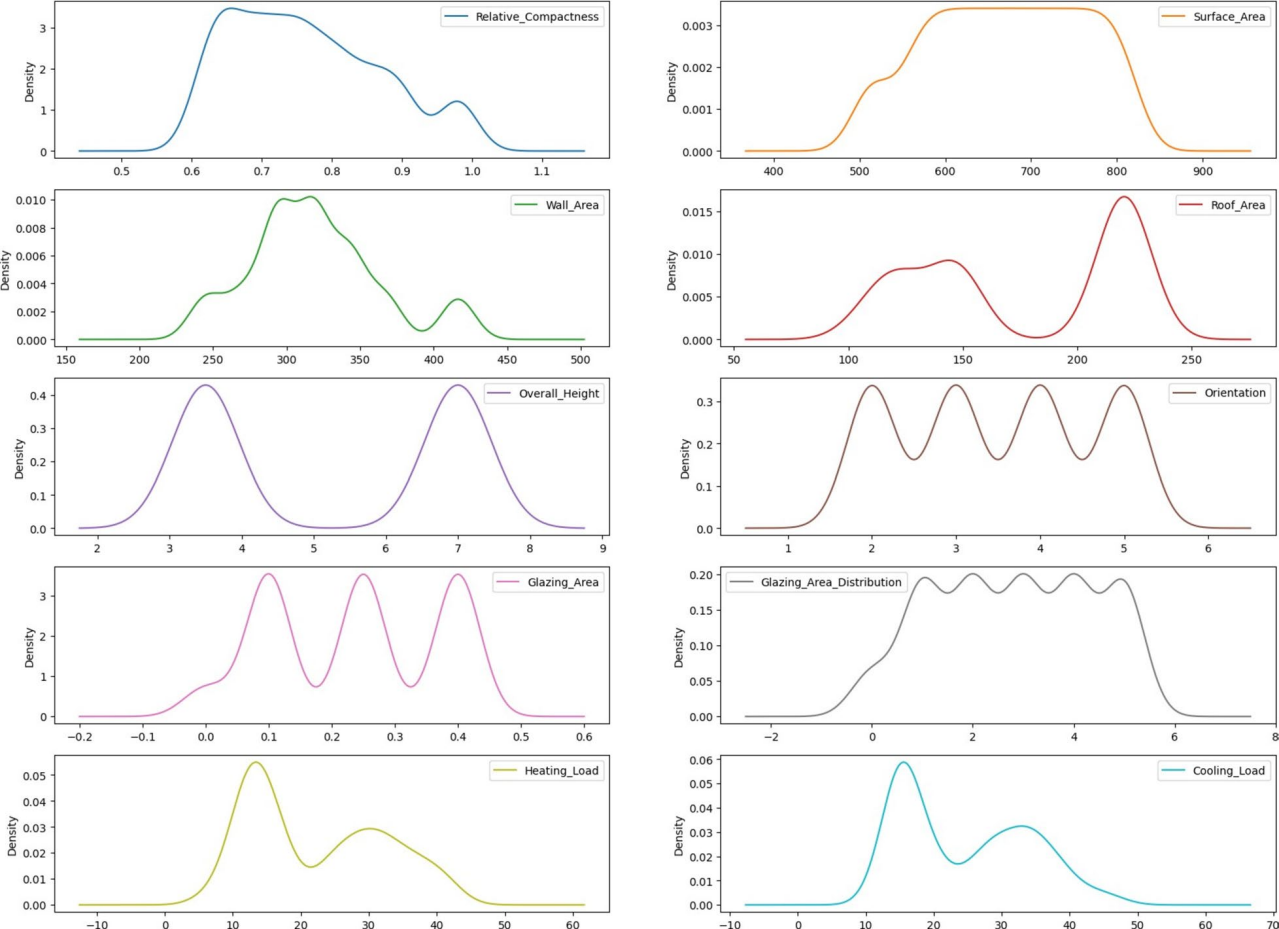
Density visualization for each energy efficiency dataset feature.

Figure 3 displays density representations for individual features from an energy efficiency dataset, along with details on their probability distribution. Each graph displays the distribution of a particular feature, with an x-axis showing the likely feature value and a y-axis representing the probability density. Higher-density regions show larger numbers of data points that correspond to specific characteristic values. By analyzing these graphics, one may determine the likelihood of seeing particular values for every parameter in the dataset, which makes it easier to understand the underlying patterns and characteristics of the data.

**Fig. 4:**
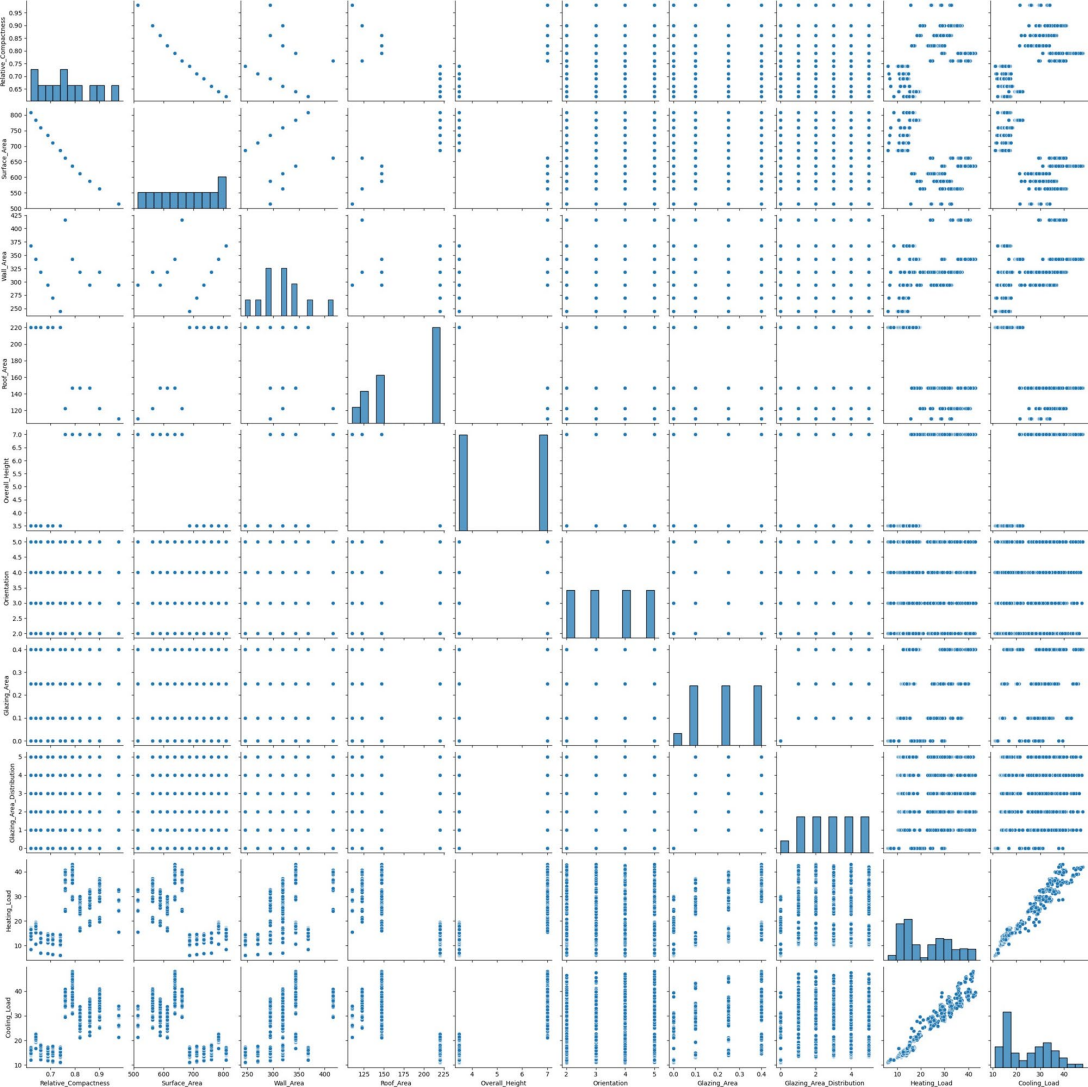
Pair plot graph for energy efficiency dataset features.

In data analysis, pair plots are a flexible visualization tool that is often used to closely analyze correlations between pairs of parameters, as demonstrated in the energy effciency dataset presented in Fig. 4. This graphic creates an organized pattern using scatterplots and histograms. A scatterplot illustrates the association among two data points, while a histogram displays the variance of just one parameter across the diagonal axis. Scatterplots are useful for showing the relationship between the variables as well as for locating anomalies, clusters, and non-linear or linear structures. Histograms are displayed diagonally and illustrate the distribution of values for every parameter, describing their shape, central tendency, and spread. Pairplots are incredibly useful for evaluating several variables at once, helping to identify patterns and formulate hypotheses.

**Fig. 5:**
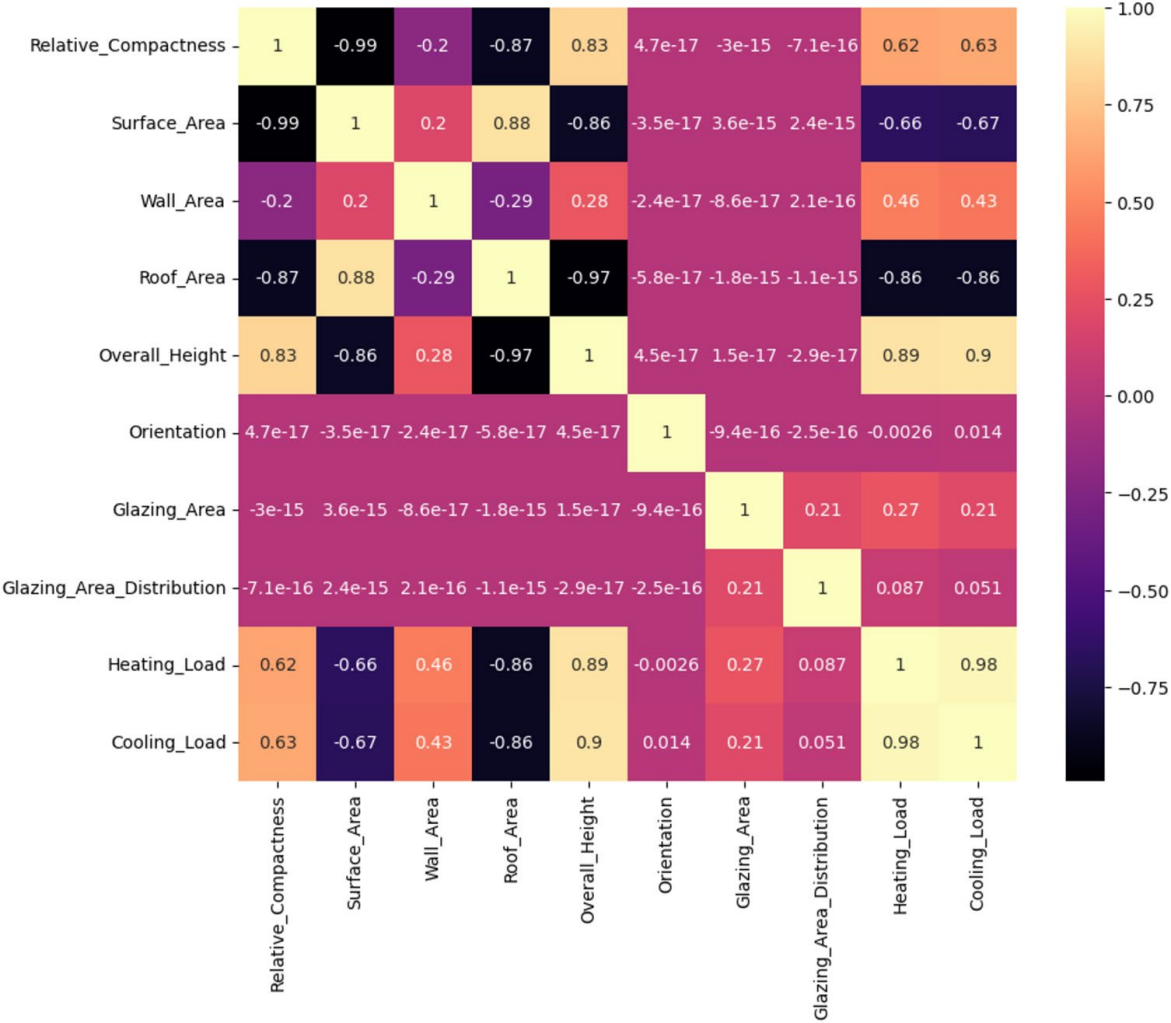
Heatmap graph for energy efficiency dataset features.

The heatmap graph in Fig. 5 shows the relationship between the heating and cooling load and different building elements. The analysis includes features including overall height, orientation, surface area, wall area, roof area, relative compactness, and glazing area and distribution. The heatmap’s cell highlights each represent the direction and degree of the association between two features. For instance, the “Heating Load” and “Relative Compactness” boxes show a weak negative association, indicating that as a building becomes more compact, the heating load reduces. On the contrary, a cell that represents the relationship between “Surface Area” and “Cooling Load” is strongly positive, suggesting that when the building’s surface area grows, it increases the cooling load. Ultimately, by assisting engineers and architects in understanding how design elements affect energy efficiency, this visualization promotes the construction of more environmentally friendly and energy-efficient structures.

**Fig. 6:**
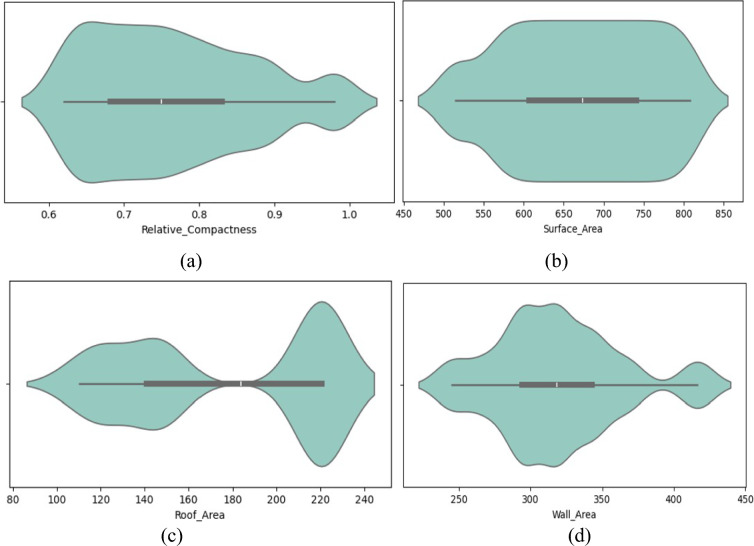
(**a**) Box plot of relative compactness feature. (**b**) Box plot of surface area feature. (**c**) Box plot of wall area feature. (**d**) Box plot for roof wall features of energy efficiency dataset.

The visualization’s box plots each show how various energy-related features are distributed across a dataset in Fig. 6. Relative compactness, surface area, wall area, and roof area are among the features shown. Each plot’s x-axis shows the range of values for that particular attribute, and the y-axis shows the relative frequency. The median is shown by the line inside the box, which itself reflects the middle quartiles of the data. Plotting of individual circles indicates that a data point is an outlier. The whiskers reach the most extreme numbers within 1.5 times the interquartile range (IQR) from the median lines. The relative compactness feature has outliers on both ends and a median value of roughly 0.7, with a range of 0.6 to 0.9. It is clear from comparing the graphs that relative compactness varies less over the dataset than surface area, wall area, and roof area.

**Fig. 7:**
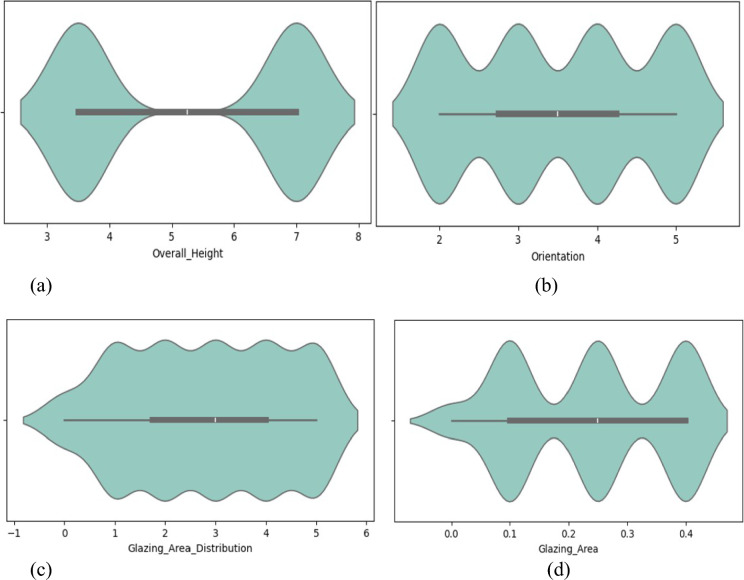
(**a**) Box plot of overall building height feature. (**b**) Orientation feature of green building box plot. (**c**) Box plot of glazing area feature of green building. (**d**) Box plot of glazing area distribution feature of green building for energy efficiency dataset.

Four features from an energy-efficient green building dataset are visualized using a box plot: Overall Height, Orientation, Glazing Area, and Glazing Area Distribution in Fig. 7. The distribution of the associated features is summarized in each box graphic. When analyzing a box plot, the median value is represented by the center line, the first and third quartiles are shown by the bottom and top of the box, respectively, and the middle 50% of the data is covered by the IQR. Each plot provides information on each attribute, including the range of values and the median value. For instance, the median height in plot (a), representing overall height, is roughly 7, with outliers on the higher end of the range between 5 and 11. Unfortunately, plots (b) and (d) lack precise x-axis labels, which makes it difficult to evaluate the data. However, orientation, glazing area, and glazing area distribution have a larger range of values, with outliers on both ends in each case.

### Data preprocessing

Data preprocessing plays a crucial role in both data assessment and the development of machine learning systems. It involves cleaning and transforming raw data to ready it for further analysis or training machine learning models. Effective preprocessing enhances the quality and efficiency of models by addressing issues such as missing data and imbalanced data. In this study, feature normalization is employed to normalize the data and data splitting, leading to improved performance of the proposed methodologies.

#### Feature normalization

Feature normalization is a preprocessing technique used to adjust the scale of feature values to a consistent range. This is particularly beneficial as many deep learning classifiers exhibit improved performance when features are within a similar range. In this study, a normalization approach is adopted to rescale dataset features between 0 and 1, promoting their uniformity and comparability^35^. Specifically, Z-score scaling, also known as standardization, is employed to transform features to have a mean of 0 and a standard deviation of 1. The mathematical expression for z-score scaling, denoted as Eq. (1), is utilized in this research.

1 $$Z_{{standardized = \frac{Z - \mu }{\sigma }}}$$

where Z is the actual attribute value, µ is the mean of the attribute in the dataset, and σ is the standard deviation of the attribute.

#### Dataset splitting

To ensure the effectiveness of machine learning models and prevent over-fitting, it’s common practice to divide the dataset into training, validation, and testing sets. This allows for assessing the models’ generalization capabilities. In this research, the dataset is divided into 70% for training and 30% for testing. The division is calculated using the following expressions:

Training datasets size

2 $${\varvec{D}}_{{{\varvec{train}} = {\varvec{round}}\left( {{\varvec{D}} \times {\varvec{trainratio}}} \right)}}$$

Test datasets size

3 $$D_{{test = D - D_{train} }}$$

Here, D represents the total number of instances in the dataset, while trainratio and test_ratio denote the proportions allocated to the training and testing sets, respectively. These equations determine the sizes of the training and test sets based on the specified ratios. Adjusting the train_ratio directly impacts the size of the training set, while the remaining instances form the test set after allocation.

### Active learning

Active Learning is an ML method that efficiently detects and recognizes data attributes using their illuminating elements. Its objective is to maximize model performance while decreasing labeling costs, notwithstanding its susceptibility to unpredictable changes.

AL selects samples with distinguishing characteristics using a well-structured framework that blends several sampling techniques, such as committee inquiry, information saturation sampling, and uncertainty sampling. Selecting a pool of unlabeled examples from the dataset is the first step in the cognitive annotation procedure for pooled-based AL. A human expert selects and annotates the most informative data points from this pool, retraining the algorithm. Until the required level of accuracy is reached, this iterative process continues. This study’s n queries and uncertainty sample dimension hyper-parameters have fixed values. While uncertainty sampling, a renowned and important selection approach, qua data electability, is used to select the number of instances to be clarified, n queries controls the frequency of model iterations. The model goes through three training processes when n_queries is set to three, with the algorithm always selecting the most informative data. The recursive procedure continues until the desired degree of prediction accuracy is attained. Two Hyperparameter that are employed in the study to control the AL process are “n_queries” and “uncertainty_sample”. The algorithm undergoes three training stages, with the amount of repetitions controlled by the “n_queries” variable. At each iteration, the system selects as numerous instructive samples as it can label. Furthermore, at the conclusion of each iteration, the selection uncertainty_sample, which is set to a query technique significance, is deemed to be the most informative. The well-known and effective uncertainty sampling technique, which selects data points that have significant electability, provides support for this choice.

By integrating these parameters and techniques, the AL algorithm repeatedly selects and labels the most pertinent samples from a repository of unlabeled data, improving the model’s efficiency. The pooled-based AL method^36^ has the following equation (4):

4 $$H\_it = modeltrainingusing \left( {L\_it, M\_it} \right)$$

5 $$W_{1} :it = git\left( {X_{1} :it} \right)$$

6 $$V_{it } = i\varepsilon v:argmaxw\varepsilon yp\left( {w/xi,wi:it} \right)$$

7 $$j_{it} = j_{it} \varepsilon argmaxj\varepsilon U_{it} H\left( {p\left( {w/xj_{it} ,y1:it - 1} \right)} \right)$$

where V_it_ indicates the set of unlabeled cases at iteration it, w_1_: for every tagged sample, it is a vector representing the model’s output, g_it_ illustrates the model that was iteratively trained on the labeled dataset, the index j_it_ designates the instance from the most instructive set, and H signifies the entropy. The suggested work uses an active and unpredictable learning methodology. Three iterations and eight regression models random forest, XGBoost, LR, KNN, GB, XGB, DT and catboost are used to calculate the active learning parameters. The method gradually expands the defined dataset while removing unknown samples by adding annotated examples from the test set. The purpose of practical performance metrics is to identify and rank critical facets of classifier performance efficiently.

### Random Forest Regressor

The Random Forest Regressor is an ensemble learning method that produces the mean forecast of each decision tree after building several of them during training. Because every decision tree in the forest has been trained using a random selection of features and training data, overfitting is minimized, and generalization is enhanced^37^. The prediction produced by a Random Forest Regressor has the following simple Eq. (8):

8 $$X^{ \wedge } = \frac{1}{M} \sum \limits_{i = 1}^{M} {\text{Ti}}\left( {\text{y}} \right)$$

where X denotes the target variable’s predicted value, the input feature’s forecasted value by M, and the prediction generated by the ith decision tree for the input feature is denoted by Ti(y). Target variable forecasted by TFR based on mean prediction of all decision trees in the forest. The forecasts are typically more accurate and dependable when using this ensemble approach than individual decision trees.

### Gradient Boosting Regressor

The Gradient Boosting Regressor is another technique for ensemble learning. It identifies a series of weak learners (typically decision trees) by fixing the errors made by the previous learner. The ultimate prediction is the sum of the predictions made by the weak learners, weighted by a learning rate^38^. Below is the equation that shows the prediction of a Gradient Boosting Regressor:

9 $$X^{ \wedge } = \frac{1}{M} \sum \limits_{i = 1}^{M} {{\gamma {\rm i Ti}}}\left( {\text{y}} \right)$$

where: X denotes the target variable’s predicted value, M is the total number of poor learners in the group. The decreasing parameter or learning rate for the i-th weak learner is denoted by γi. The i-th weak learner (tree)’s prediction for the input characteristics x is Ti(x). The Gradient Boosting Regressor incorporates the predictions of several weak learners, each of whom is trained to rectify the mistakes of the preceding ones and predict the target variable. The learning rate determines the amount that each poor learner contributes to the final prediction.

### K Neighbors Regressor

The K Nearest Neighbors Regressor (KNN Regressor) is a supervised learning technique created especially for regression tasks. The desired value for a particular data point can be predicted by aggregating the target values of that data point’s closest neighbors in the range of features. The process involves calculating the distance between each target value and all the additional information in the training dataset, usually with a distance measure like the Euclidean distance. The k nearest data points, or neighbors, are identified as the target data points based on these distances. The target values of these k nearest neighbors are averaged to determine the predicted desired value for the regression analysis^38^

### Cat Boost Regressor

The CatBoost Regressor is a gradient-boosting method created especially for regression challenges; it forecasts uninterrupted numerical values. It constructs a series of decision trees, one after the other, in order to produce forecasts. The input space is subdivided into smaller regions with each decision tree, and a constant value is assigned to each sector for prediction. catboost Regressor is an algorithm that continuously improves predictions by gradient boosting, maximizing a chosen loss function, and incorporating new trees to correct errors caused by previous ones. Catboost efficiently handles chaotic and lacking information in data by employing robust gradient-based learning.

#### Extreme Gradient Boosting Regressor

The Extreme Gradient Boosting (XGBoost) Regressor is a ML technique that is restricted to problems involving regression. It functions exceptionally well and effectively. As part of the boosting approach family, XGBoost constructs a sequence of weak learners, often decision trees, and then iteratively improves predictions by decreasing mistakes from previous iterations. XGBoost performs gradient-based optimization within the GB paradigm by employing first- and second-order gradients to repeatedly improve the model’s efficiency.

### Linear regression

The statistical technique known as linear regression involves fitting a linear equation to observed data in order to model the connection between a dependent variable and a few independent variables. The basic premise of linear regression is that there is a linear relationship between the independent variable or variables and the dependent variable.

#### Light GBM Regressor

Light GBM Regressor is one of the most well-known members of the boosting algorithm family; it is very efficient and adept at managing large datasets. As opposed to building trees depth-wise, Light GBM develops trees leaf-wise, which often yields higher efficiency and deeper trees with fewer nodes, especially when working with huge datasets. To further speed up processing on multi-core CPUs and GPUs, Light GBM has native support for categorical traits, multiple regularization strategies to avoid overfitting, and the ability to leverage parallel and GPU learning systems.

### Decision Tree Regressor

The DT Regressor is a versatile ML technique for forecasting continuous target variables based on input data. It divides the input space recursively into smaller segments, assigning a forecasted target value to each one. The model’s structural design is akin to a tree, with leaf nodes signifying anticipated outcomes and core nodes representing judgments based on feature values. The algorithm iterates until the stopping conditions are satisfed by choosing features and thresholds to reduce variation within data subsets during training. Decision rules are followed when one navigates the tree from root to leaf to make predictions for new instances^38^.

Table 2 the proposed Algorithm 1 is an active learning pipeline using machine learning methods to classify text input according to its intended use. Data preprocessing, which includes normalization, is the first step in the procedure. Energy efficiency data is used as input, and a sequence of procedures is applied to train and evaluate multiple regression models, such as the Random Forest, Gradient Boosting, XGBoost, Linear, K-Nearest Neighbors, CatBoost, LightGBM, and Decision Tree regressions. Allocating training and testing datasets, setting output parameters for prediction accuracy, and initializing iteration counts and query techniques. The algorithm initializes an active learner instance and performs active learning rounds for every regression model. In these iterations, the model is changed in accordance with the selection of uncertain samples from the test data and the queries made to the active learner for predictions. Ultimately, the algorithm displays overall performance metrics for each regression model along with the final predictions after assessing the model’s performance using predefined metrics.

**Table 2:** Proposed algorithm of predicting energy efficiency in green building using AL.

**Algorithm 1** Proposed Algorithm of Predicting Energy Efficiency in Green Building using AL
1: **Input** Energy Efficiency Dataset labels
2: *Cit* = 3 {Count of Iterations}
3: *Qstrategy* = *USample* {Query Strategy = Uncertain sample}
4: **Output** Energy Efficiency Prediction in Green Building
5: *EMeasure* : Prediction Accuracy (*R*2), MSE, MAE,RAE
6: Initialization ’a’
7: List of Regressor: RFR, GBR, XGBoostR, LR, KNR, CatBoostR, LightBGMR and DTR
8: ’Alg_names’ indicates the name, and ’algorithms’ describe the list
9: Assign the training and testing groups the appropriate variables, ’X_train’, ’X_test’, ’y_train’, and ’y_test’
10: Divide the dataset into sets for testing and training
11: **for** Algo in Algos **do**
12: **Assign the following initialization variables to the Active Learner:**
13: Pass the model, X_train, and y_train points to the respective arguments to create an Active Learner object 14: Generate a custom_query_strategy technique called Y_pool that accepts the set of unlabeled cases as input
15: The function reinstate the uncertainty_sampling with the arguments learner, Y_pool
16: Set ’m_round’ to 3 and provide the value "active learning iterations."
17: **for** Round in span(m_round) **do**
18: Imprint "round. (iteration + 1)"
19: Call "custom_query_strategy" with "Y_test" as the varaible and assign the results to "query_idx"
20: Select "X_test" and "y_test" for indices in "query_idx"
21: X_pool and y_pool ought to be assigned the fnal values in accordance with
22: Consider the predictions for the most uncertain situations in the "X_pool" as an input
23: Assign a value to the variable "y_pred_pool"
24: With the inputs "X_pool" and "y_pred_pool," To the "learner" object, add the unclear cases along with their expected labels
25: **end for**
26: Display the regressor assessment metrics, containing the average accuracy (*R*2), MSE, MAE, and RAE
27: Provide the final classification results for each classifier
28: **end for**

## Result and discussion

This section delves deeper into the application of an AL method that integrates machine learning methods for energy consumption in green buildings on the energy consumption dataset. Training uses 70% of the dataset, while testing uses 30%. With the dataset provided, this model learns through the use of the ML regression model. This model’s performance indicator includes, but are not limited to, prediction accuracy (R^2^), MSE, MAE, and RAE. Each of these metrics demonstrates the process’s effectiveness. This part summarizes and grades the test results as well as provides a thorough and insightful analysis of them.

This study experimented with a pre-selected collection of tools and technologies. Python 3.8.8, a highly influential programming language in the field of ML, offers numerous modules and tools to expedite complex data processing, analysis, and visualization. The modest conductor is Jupyter Notebook, a development platform widely recognized for its exceptional ability to supply an ideal programming environment that facilitated the creation of Python 3.8.8. Because of its remarkable stability and efficiency, Windows is the best operating system in this scenario since it enables Python apps to run concurrently and cooperatively. Installing this architecture is made easier by the fact that the mainframe is a Dell Core i5 laptop. Even with a powerful CPU and significant memory requirements, it operated efficiently in high-performance mode.

### Evaluation metrics

This research evaluates the proposed model’s efficiency using a wide range of significant assessment criteria, all of which offer useful information about the model’s operation. Several measures are frequently utilized as standards for assessing performance, such as prediction accuracy (R^2^), MSE, MAE, and RAE. The following Eq. (10) makes this simple to understand. Even though the measure’s calculation is simple, it has a significant impact.

Prediction Accuracy (R^2^): The prediction accuracy in a regression model quantifies the percentage of variance in the dependent variable (target) that can be predicted from the independent variables (features). This is often expressed as the coefficient of determination (R^2^). Generally, it has a range of 0–1, with 1 denoting an exact forecast and 0 denoting no predictive ability. The following formula (10) is used to determine the R^2^ value:

10 $$R^{2} = {\raise0.7ex\hbox{${SSres}$} \!\mathord{\left/ {\vphantom {{SSres} {SStot}}}\right.\kern-0pt} \!\lower0.7ex\hbox{${SStot}$}}$$

where: The regression model does not account for the total variance, which is measured by *SSres* or the sum of squares of the residuals and The total sum of squares *SStot* calculates the dependent variable’s overall variance.

***Mean Squared Error:*** The Mean Squared Error (MSE) measures the average squared divergence between the expected and actual numbers. This formula (11) is used to compute it:

11 $$MSE = \frac{1}{n}\mathop \sum \limits_{i = 1}^{n} \left( {{\text{Xi}} - {\text{X}}^{ \wedge } {\text{i}}} \right)^{2}$$

where: The quantity of observations is n. Xi represents the target variable’s real value for observation. Xˆi stands for the target variable’s anticipated value in the observation.

***Mean Absolute Error:*** The average absolute difference between the expected and actual values is measured by the Mean Absolute Error (MAE).

The following Eq. (12) is employed to compute it:

12 $$MAE = \frac{1}{n}\mathop \sum \limits_{i = 1}^{n} \left| {{\text{Xi}} - {\text{X}}^{ \wedge } {\text{i}}} \right|^{2}$$

where: The quantity of observations is m. Xi represents the target variable’s real value for observation. Xˆi stands for the target variable’s anticipated value in the observation, and |.| indicates the absolute value.

***Relative Absolute Error:*** The average relative variance between the expected and actual values is measured by the Relative Absolute Error (RAE). The following formula (12) is utilized for calculating it:

13 $$MAE = \frac{1}{n}\mathop \sum \limits_{i = 1}^{n} \frac{{\left| {{\text{Xi}} - {\text{X}}^{ \wedge } {\text{i}}} \right|}}{{\left| {{\text{Xi}} - {\text{X}}^{ - } {\text{i}}} \right|}}$$

where: The number of observations is n. Xi represents the target variable’s real value for observation. Xˆi stands for the target variable’s anticipated value in the observation, and X¯i symbolizes the average of the real values found in all of the observations; the Absolute value is represented by |.|.

### Results of the proposed approach

The study evaluated the mean squared error (MSE), mean absolute error (MAE), relative absolute error (RAE), and prediction accuracy (R^2^) for energy consumption in green buildings using the energy efficiency dataset. The models included eight active learning-based regression models: RFR-AL, GBR-AL, KNR-AL, CBR-AL, XGBR-AL, LR-AL, LGBMR-AL, and DTR-AL. Three iterations are examined to calculate the prediction accuracy (R2) and error rates of each regressor.

The results of regression models that were assessed using several metrics to forecast the target variable Y1 (heating_Load) are shown in Table 3.

**Table 3:** Result based on regression metrics for target variable Y1 (Heating_Load).

Regression model	*R*^2^	MSE	MAE	RAE
RFR-AL # 1	0.9950	0.5445	0.3887	0.0171
RFR-AL #2	0.9947	0.5726	0.4011	0.0176
RFR-AL # 3	0.9951	0.5287	0.4045	0.0178
GBR-AL # 1	0.9907	1.0092	0.6424	0.0282
GBR-AL #2	0.9907	1.0092	0.6425	0.0282
GBR-AL # 3	0.9907	1.0092	0.6424	0.0282
KNR-AL # 1	0.9700	3.2378	1.1017	0.0484
KNR-AL #2	0.9700	3.2378	1.1017	0.0484
KNR-AL # 3	0.9700	3.2378	1.1017	0.0484
CBR-AL # 1	0.9975	0.2667	0.2984	0.0131
CBR-AL #2	0.9975	0.2667	0.2984	0.0131
CBR-AL # 3	0.9975	0.2667	0.2984	0.0131
XGBR-AL # 1	0.9941	0.6414	0.3774	0.0166
XGBR-AL #2	0.9941	0.6414	0.3774	0.0166
XGBR-AL # 3	0.9941	0.6414	0.3774	0.0166
LR-AL # 1	0.9666	3.6108	1.2413	0.0545
LR-AL #2	0.9666	3.6108	1.2413	0.0545
LR-AL # 3	0.9666	3.6108	1.2413	0.0545
LGBMR-AL # 1	0.9936	0.6904	0.5035	0.0221
LGBMR-AL #2	0.9936	0.6904	0.5035	0.0221
LGBMR-AL # 3	0.9936	0.6904	0.5035	0.0221
DTR-AL # 1	0.9948	0.5634	0.3569	0.0157
DTR-AL #2	0.9943	0.6149	0.3827	0.0168
DTR-AL # 3	0.9899	1.0896	0.4192	0.0184

Key: R^2^, prediction accuracy; MSE, mean squared error; MAE, mean absolute error; RAE, relative absolute error.

According to the findings, a Random Forest Regressor with specific variables provides a high prediction accuracy (R^2^) of 0.9950 for iteration 1, suggesting a significant connection between the predicted and actual values. Its average errors are modest, as indicated by its MSE of 0.5445 and MAE of 0.3887. The RAE of 0.0171 also indicates a low relative error in relation to the true value. Similar models with slightly different error rates, such as those at iterations 2 and 3, continue to retain excellent R^2^ (0.9947 and 0.9951).

Gradient boosting Regressor (GBR) models exhibit excellent R^2^ even with somewhat higher error rates than the RFR, despite a modest decrease in R^2^ (0.9951) at iteration 1. Active learning-based KNR (KNR-AL) obtained the best R^2^ of 0.9700, MSE of 3.2378, and MAE of 1.1017 with RAE of 0.0484 for all three iterations. The prediction accuracy (R^2^) for the CBR (CatBoost Regressor) is 0.9975, suggesting a very high rate of correlation between the predicted and actual values. MSE of 0.2667 indicates extremely low average squared errors between expected and actual values. The MAE of 0.2984 indicates a relatively low absolute error on average between the anticipated and actual values. The value of RAE in three iterations is 0.0131, which is very little error. The Extreme Gradient Boosting Regressor-Active Learning (XGBR-AL) attained a prediction accuracy (R^2^) with a value of 0.9941, MSE value of 0.6414, MAE value of 0.3774, and RAE value of 0.0166. The proposed XGBR-AL model result shows that consistently across a range of all iterations. The LR-AL model delivers performance in terms of Prediction Accuracy with a value of 0.9666, MSE with a value of 3.6108, MAE with a value of 1.2413, and RAE with a value of 0.0545 in all three iterations. LGBMR-AL models have the subsequent values for Prediction Accuracy: (R^2^) of 0.9936, MSE of 0.6904, MAE of 0.5035, and RAE of 0.0221 for every iteration.

Active learning-based DTR Prediction Accuracy (R^2^) ranges from 0.9899 to 0.9948, suggesting a strong correlation between the expected and observed values. The MSE range from 0.5634 to 1.0896 indicates the average error between the squared values predicted and those observed. The average absolute discrepancy between the expected and actual numbers is represented by the MAE, which varies from 0.3569 to 0.4192. The RAE has a range of 0.0157 to 0.0184, indicating different degrees of relative error with respect to the true value.

The results of regression models that were assessed using several metrics to forecast the target variable Y2 (Cooling_Load) are displayed in Table 4. For all three rounds, the active learning-based RFR R^2^ ranged from 0.9757 to 0.9765, the MSE from 2.1775 to 2.2447, the MAE from 0.8087 to 0.8351, and the RAE from 0.0325 to 0.0336. The active learning-based GBR indicates that R^2^ ranges from 0.9772 to 0.9776, MSE ranges from 2.0761 to 2.1077, MAE ranges from 0.8712 to 0.8765, and RAE ranges from 0.0351 to 0.0353 for each of the three rounds.

**Table 4:** Result based on regression metrics for target variable Y2 (Cooling_Load).

Regression model	*R*^2^	MSE	MAE	RAE
RFR-AL # 1	0.9757	2.2447	0.8351	0.0336
RFR-AL #2	0.9758	2.2432	0.8293	0.0334
RFR-AL # 3	0.9765	2.1775	0.8087	0.0325
GBR-AL # 1	0.9774	2.0945	0.8743	0.0352
GBR-AL #2	0.9776	2.0761	0.8712	0.0351
GBR-AL # 3	0.9772	2.1077	0.8765	0.0353
KNR-AL # 1	0.9498	4.6474	1.3006	0.0524
KNR-AL #2	0.9498	4.6474	1.3006	0.0524
KNR-AL # 3	0.9498	4.6474	1.3006	0.0524
CBR-AL # 1	0.9883	1.0869	0.5869	0.0236
CBR-AL #2	0.9883	1.0869	0.5869	0.0236
CBR-AL # 3	0.9883	1.0869	0.5869	0.0236
XGBR-AL # 1	0.9798	1.8732	0.7444	0.0300
XGBR-AL #2	0.9798	1.8732	0.7444	0.0300
XGBR-AL # 3	0.9798	1.8732	0.7444	0.0300
LR-AL # 1	0.9584	3.8463	1.4451	0.0582
LR-AL #2	0.9584	3.8463	1.4451	0.0582
LR-AL # 3	0.9584	3.8463	1.4451	0.0582
LGBMR-AL # 1	0.9756	2.2564	0.9891	0.0398
LGBMR-AL #2	0.9756	2.2564	0.9891	0.0398
LGBMR-AL # 3	0.9756	2.2564	0.9891	0.0398
DTR-AL # 1	0.9617	3.5461	0.9135	0.0368
DTR-AL #2	0.9611	3.6014	0.9104	0.0366
DTR-AL # 3	0.9590	3.7957	0.9343	0.0376

Key:* R*^*2*^, prediction accuracy; MSE, mean squared error; MAE, mean absolute error; RAE, relative absolute error.

The Prediction Accuracy (R^2^) for each of the three iterations is 0.9498, the MSE is 4.6474, the MAE is 1.3006, and the RAE is 0.0524 of KNR-AL. Likewise, for CB-AL, the R^2^ indicates a strong degree of correlation between the projected and actual values, at 0.9883. With an MSE of 1.0869, the average squared error between the anticipated and actual values is quite small. With an MAE of 0.5869, the average absolute error between the expected and actual values is rather small. RAE is 0.0236, indicating that each iteration’s relative error to the actual value is low. Predictive accuracy (R^2^) for XGBR-AL is 0.9798, MSE is 1.8732, and MAE is 0.7444 for each of the three rounds. The prediction Accuracy (R2) for LR-AL is 0.9584, the MSE and MAE are 3.8463 and 3.8463, respectively, and the RAE is 0.0582 for every iteration. The predicted Accuracy (R^2^) for LGBM-AL is 0.9756, the MSE is 2.2564, the MAE is 0.9891, and the RAE is 0.0398 for every iteration. Similarly, the Prediction Accuracy (R^2^) for DTR-AL ranges from 0.9590 to 0.9617, suggesting a reasonably high level of correlation between the expected and actual results. The MSE ranges from 3.5461 to 3.7957, indicating different average degrees of squared errors between the expected and actual values. The MAE shows different amounts of absolute errors on average between the predicted and actual values, ranging from 0.9104 to 0.9343. The RAE ranges from 0.0366 to 0.0376, indicating different degrees of relative error in relation to the actual number. DTR-AL error values vary from iteration to iteration. In iteration 1, the error value is less than in iteration 3, and the DTR-AL model performs slightly differently depending on the parameter.

Table 5 provides the prediction accuracy (R^2^) of two classes, Y1 (heating load) and Y2 (cooling load), of an ML regression model. The prediction accuracy attained by RFR-AL is 0.9951 for Y1 and 0.9765 for Y2. Y2 performance is better than Y1 class of RFR-AL model, in contrast GBR-AL achieve 0.9700 for Y1 and 0.9776 for Y2 that is almost same for both class. CBR-AL is performed well as compared all proposed model with value attained 0.9975 for Y1 and 0.9883 for Y2. The prediction accuracy attained by XGBR-AL is 0.9941 for Y1 and 0.9798 for Y2. Y2 performance is lower than Y1 class of XGBR-AL model, in contrast LR-AL achieve 0.9666 for Y1 and 0.9584 for Y2 in which Y1 surpass the performance than Y2. LGBMR-AL attained 0.9936 for Y1, which is higher than the Y2 class. That value is 0.9756. DTR-AL attained 0.9948 for Y1, which is also higher than the Y2 class, whose value is 0.9617.

**Table 5:** Average prediction accuracy (R^2^) comparison of variable Y1 (Heating_Load) and Y2 (Cooling_Load).

Regression model	Y1 (Prediction accuracy *R*^2^)	Y2 (Prediction accuracy *R*^2^)
RFR-AL	0.9951	0.9765
GBR-AL	0.9700	0.9776
KNR-AL	0.9700	0.9498
CBR-AL	0.9975	0.9883
XGBR-AL	0.9941	0.9798
LR-AL	0.9666	0.9584
LGBMR-AL	0.9936	0.9756
DTR-AL	0.9948	0.9617

### Discussion and limitation

The proposed model enhances environmental sustainability in green buildings by optimizing energy consumption through accurate predictions of heating and cooling needs, integrating with Building Automation Systems (BAS) to improve operational efficiency, and reducing carbon footprints by minimizing energy waste. Currently, we have not yet applied the proposed model in specific case studies. However, we plan to implement it in various green building projects in the near future. By applying the model to diverse settings such as office buildings, residential complexes, and university campuses, we aim to validate its effectiveness in optimizing energy consumption and enhancing sustainability. These future implementations will provide concrete examples and further demonstrate the model’s practical impact on reducing energy waste and improving environmental performance in green buildings.

The proposed CBR-AL (CatBoost Regressor with Active Learning) model offers notable improvements over existing predictive models for green building energy consumption. It achieves higher accuracy, with values of 0.9975 for cooling and 0.9883 for heating. The integration of active learning enhances efficiency by focusing on the most informative data, reducing the need for extensive labelling. Cat Boost’s robustness ensures effective handling of complex data, while active learning improves generalization and adaptability to changing conditions. Despite its computational demands, the model balances performance and efficiency, making it suitable for real-time Building Automation Systems (BAS). Its ability to scale, adapt to external factors and continuously improve through dynamic learning positions it as a powerful tool for optimizing energy efficiency and sustainability in green buildings. The paper uses MSE, MAE, RAE, and R^2^ to assess model performance comprehensively. MSE highlights larger errors more than smaller ones, making it useful for understanding overall error magnitude. MAE offers a straightforward average error measurement without emphasizing large deviations. RAE compares MAE to a baseline model, showing the model’s relative improvement. R^2^ indicates how well the model explains the variance in the target variable. Together, these metrics provide a detailed evaluation of accuracy, error magnitude, and explanatory power.

The proposed model, while promising, has several limitations. Its accuracy depends on data quality, and incomplete or noisy data can impair performance. The computational intensity of the active learning-based approach challenges real-time applications or buildings with limited resources. Integration with existing Building Automation Systems (BAS) may require significant adjustments, leading to compatibility issues and additional costs. The model may struggle with unpredictable energy usage due to occupant behavior variability. Scalability is a concern, as maintaining accuracy in larger or multiple buildings is challenging. External factors like extreme weather, energy price changes, or unforeseen events (e.g., pandemics) can impact energy consumption patterns, which the model might not fully account for. The initial setup and calibration phase can be time-consuming and may require expert intervention. Regular updates and maintenance are necessary to address changes in building usage, occupancy patterns, or energy systems. Acknowledging these limitations allows for targeted improvements, ensuring the model delivers its intended benefits in green building applications.

## Conclusion

This study highlights significant findings in leveraging artificial intelligence for green building energy consumption. By utilizing an active learning-based machine learning model, the research has developed an effective predictive approach to enhance energy efficiency in green buildings. The proposed CBR-AL model demonstrated exceptional prediction accuracy, achieving values of 0.9975 for cooling and 0.9883 for heating. This performance surpasses traditional machine learning models and underscores the model’s ability to reduce energy consumption while improving indoor environmental quality significantly. The incorporation of data visualization and preprocessing techniques further strengthened the model’s reliability, proving its effectiveness in advancing environmental sustainability within green building design.

The ripple effects of this research extend beyond improved energy efficiency. The successful application of the CBR-AL model paves the way for future advancements in sustainable architecture, including substantial cost savings and reduced carbon footprints. The model sets a new benchmark for predictive modelling in energy management, which can influence green building practices on a broader scale. Future research could build on these fndings by incorporating diverse datasets, exploring advanced modelling techniques, integrating real-time data, and enhancing feature engineering. Expanding the application domains and fostering collaboration with stakeholders will further drive the development of innovative solutions for energy-efficient and environmentally sustainable building designs.

## Data Availability

The datasets used during the current study are available online in the Kaggle Repository (https://www.kaggle.com/datasets/elikplim/eergyeffciency-dataset).
